# Exercise therapy improves aerobic capacity of inpatients with major depressive disorder

**DOI:** 10.1002/brb3.469

**Published:** 2016-04-22

**Authors:** Arno Kerling, Anne von Bohlen, Momme Kück, Uwe Tegtbur, Lena Grams, Sven Haufe, Elke Gützlaff, Kai G. Kahl

**Affiliations:** ^1^ Institute of Sports MedicineHannover Medical SchoolCarl‐Neuberg‐Str. 130625HannoverGermany; ^2^ Department of Psychiatry, Social Psychiatry and PsychotherapyHannover Medical SchoolCarl‐Neuberg‐Str. 130625HannoverGermany; ^3^ Institute of Clinical PharmacologyHannover Medical SchoolCarl‐Neuberg‐Str. 130625HannoverGermany

**Keywords:** Aerobic capacity, major depression, maximum oxygen consumption, physical training, ventilatory anaerobic threshold

## Abstract

**Background:**

Unipolar depression is one of the most common diseases worldwide and is associated with a higher cardiovascular risk partly due to reduced aerobic capacity.

**Objectives:**

Therefore, the aim of our study was to examine whether a structured aerobic training program can improve aerobic capacity in inpatients with MDD (major depressive disorder).

**Methods:**

Overall, 25 patients (13 women, 12 men) diagnosed with MDD were included in the study. Parameters of aerobic capacity, such as maximum performance, maximum oxygen consumption, and VAT (ventilatory anaerobic threshold), were assessed on a bicycle ergometer before and 6 weeks after a training period (three times per week for 45 min on two endurance machines). In addition, a constant load test was carried out at 50% of the maximum performance prior to and after the training period. The performance data were compared with 25 healthy controls matched for sex, age, and body mass index before and after the training period.

**Results:**

Compared to controls, patients with MDD had significantly lower aerobic capacity. After training, there was a significant improvement in their performance data. A significant difference remained only for VAT between patients with MDD and healthy controls.

**Conclusion:**

With regard to the coincidence of MDD with cardiovascular and cardiometabolic disorders, a structured supervised exercise program carried out during hospitalization is a useful supplement for patients with MDD.

## Introduction

Many neurological and psychiatric diseases are characterized by reduced physical activity and capacity; however, in recent years there have been encouraging studies that show positive effects of regular physical activity on these symptoms (Malchow et al. 2014; Motl and Sandroff 2015).

Unipolar depression is one of the most frequent illnesses worldwide with a lifetime prevalence of approximately 17% in western countries. It is assumed that unipolar depression will represent the second most common disease after cardiovascular diseases in the year 2030, which is still more common than tumor diseases (Spaner et al. 1994; Mathers and Loncar 2006; Ferrari et al. 2013)**.** Patients with depression exhibit an increased cardiovascular mortality and morbidity**,** behaviors associated with an unhealthy lifestyle (inactivity, smoking, unhealthy eating behavior) (Hare et al. 2014)**,** an increased incidence of metabolic syndrome (Kahl et al. 2012; Vancampfort et al. 2014), increased intra‐abdominal and pericardial fat content (Greggersen et al. 2011; Kahl et al. 2014)**,** increased hypercortisolism (Stetler and Miller 2011), and dysregulation of proinflammatory cytokines (Dowlati et al. 2010)**.**

A population‐based study in Western Australia found a gap in life expectancy in patients with depressive disorder which was growing in the last decades and was round about 15 years less for male and 12 years less for female‐depressed patients in 2005. Around 30% of excess deaths were attributable to cardiovascular disease in women (Lawrence et al. 2013).

A review from Josefsson T indicates that a physical exercise intervention in depressive disorders has a moderate to large antidepressant effect (Joseffson et al. 2014) and it is discussed that exercise may be even more effective in clinically depressed people (Rethorst et al. 2009). Another review concluded that exercise interventions may have a small short‐term antidepressant effect as long as the exercise intervention is carried out (Krogh et al. 2011).

Physical activity patterns of men and women affected by depressive and anxiety disorders show large amounts of sedentary time, and recommendations of activity guidelines are not fulfilled in most of these patients (Helgadottir et al. 2015).

Long‐term inactivity leads to a reduced aerobic capacity, which represents an important predictor of cardiovascular mortality (Dhoble et al. 2014). Some authors like Blair (2009), considered cardiopulmonary efficiency as an even more important indicator of lifetime risk than the classical cardiovascular risk factors. Considering these findings, it is particularly important for patients with depression to increase their aerobic capacity. Furthermore, there is a connection between reduced physical activity and the presence of depressive symptoms, in that both entities mutually and negatively affect each other (Azevedo Da Silva et al. 2012). The meta‐analysis from Josefsson also shows that very few exercise studies used a defined exercise dose and provided guided exercise (Joseffson et al. 2014).

Therefore, the aim of this study was to examine the effects of a structured, supervised training program accomplished during hospitalization on the improvement of aerobic capacity in inpatients with MDD (major depressive disorder).

## Patients and Methods

The study was approved by the local ethics committee; all participants were informed about the possible risks and submitted their written consent before inclusion in the study. Twenty‐five depressed inpatients (13 women, 12 men) in the age range between 18 and 65 years were included in the study. Patients were matched with a healthy control group for age, gender, and BMI (body mass index), and performance data were compared between groups. The control group was examined in the context of an outpatient sports medicine health investigation. Exclusion criteria for both groups were regular participation in exercise activities during the last 6 months (at least one hour per week), present cardiovascular illnesses, type I or type II diabetes mellitus, acute or chronic infection illnesses, tumors, pregnancy, anemia, schizophrenia, and substance use disorders, as well as orthopedic and neurological diseases that limit the implementation of bicycle ergometry.

### Assessment of major depression

A diagnosis of major depression was based on the criteria of the DSM‐IV (Diagnostic and Statistical Manual of Mental Disorders) (American Psychiatric Association, 1994). All patients underwent the SCID (Structured Clinical Interview) for DSM‐IV Axis I Disorders, administered by experienced psychiatrists. The SCID is an expert interview for clinicians, and is seen as “gold standard” in the diagnostic process of psychiatric disorders. According to the results of the interview, all patients fulfilled the diagnostic criteria for major depressive disorder. Among the 25 patients included, 19 (76%) received antidepressant pharmacotherapy (selective serotonin reuptake inhibitors, SSRI, and selective serotonin and noradrenaline reuptake inhibitors, SSNRI).

### Spiroergometry

For testing maximum oxygen uptake (VO_2peak_), participants performed an incremental exercise test under the supervision of a physician using a spirometric system (Viasprint 150p, Ergoline GmbH, Bitz, Germany) on a speed‐independent bicycle ergometer (Ergometrics 900 sec, Ergoline). ECG (Electrocardiogram)‐monitoring was performed with the subject in a sitting position. The incremental test started with a load of 20 W at a constant cadence of 60–70 revolutions per minute, and the load increased 10 W every minute until the onset of subjective overexertion (peripheral muscle fatigue or dyspnea). The subjective perceived exertion was assessed by the Borg scale, ranging from extremely light to extremely hard (Borg 1998). The same test protocol was used after the training period. Heart rate and oxygen uptake were continuously measured breath by breath and averaged over 10 sec intervals. Blood pressure and blood lactate concentration were acquired at rest, 1 min after the start of testing and every 3 min during the test. Capillary blood samples of 20 *μ*L were taken from the arterialized earlobe, deproteinized, and then measured with a lactate analyzer (Ebio 6666, Eppendorf, Berlin, Germany).

As parameters of aerobic capacity, maximum oxygen consumption (VO_2peak_), VAT (ventilatory anaerobic threshold), and the maximum power, were measured absolutely and in relation to the body weight. As a conversion factor for the MET (metabolic equivalent) related to the weight‐referred VO_2peak_ **,** we used 3.5 mL/min/kg for men and 3.15 mL/min/kg for women.

### Constant load test

Two to five days after spiroergometry, a constant load test on a bicycle ergometer under ECG‐monitoring at 50% of the maximally reached achievement was performed for the patients with MDD.

The test included a 6‐min warm‐up phase, a 20‐min training phase, and a 4‐min recovery phase. To confirm that the chosen intensity did not lead to an overload, capillary blood samples taken from the ear lobe were examined for a lactate steady state, which indicates the absence of the overload. The capillary samples were taken at rest, after the warm‐up phase (after 6 min), and three times during the last ten minutes (at minute 16, 21, and 26) of the exercise phase. An increase in blood lactate of not more than 0.5 mmol/L between minute 16 and 26 was defined as a lactate steady state. The appropriate achievement was then used in future sessions in the training program. The test was repeated at the end of the training phase under identical conditions.

### Exercise therapy

During inpatient treatment, three training units were performed per week, with each session lasting 45 min (with at least 1 day break between the units), for 6 weeks. The training program started with a 25‐min unit on a bicycle ergometer at 60–70 revolutions per minute. Blood pressure should not have exceeded 180/100 mmHg, and subjective perceived exertion on the Borg scale should have been 13–14 at maximum. The second workout phase was performed directly after cycling. The endurance training was performed for 20 min and could be continued on a cross‐trainer, a stepper, an arm ergometer, a treadmill, a recumbent ergometer, or a rowing ergometer, as preferred by the participant. Heart rate was continuously monitored via ECG. The training heart rate was allowed to be a maximum of 10% above the average heart rate on the bicycle ergometer for all devices, except for the recumbent ergometer (same heart rate) and the arm ergometer (heart rate should be approximately 10% lower). The workload was increased by 10% when heart rate and exertion on the Borg scale decreased by a predetermined amount and blood pressure did not exceed 180/100 mmHg.

### Statistics

All data are presented as the mean ± standard deviation. Data were tested for a normal distribution using the Kolmogorov–Smirnov test. The group comparisons between patients and controls were accomplished with two‐sided unpaired Student *t*‐tests for parametric data or with the Mann–Whitney *U*‐test for nonparametric data. The equality of the variances was tested with the Levene's test for normally distributed data. Cohen's *d* was calculated for the effect size. Comparisons of the patient data before and after the exercise program were performed with two‐sided paired *t*‐tests for parametric data and Wilcoxon tests for nonparametric data. Significance was accepted at *P < *0.05. All tests were performed with SPSS version 22 (IBM Corp., Armonk, NY).

## Results

**Figure 1:**
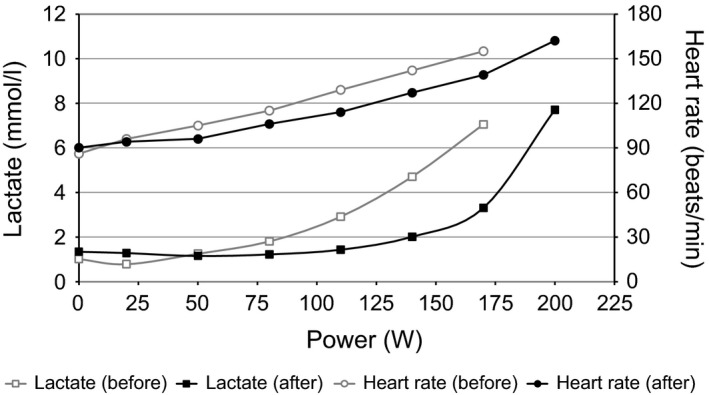
Example of the right shift of the lactate performance curve in a patient after exercise therapy. In the second test, there is a higher total output, a smaller lactate score, and a smaller heart rate within an identical test setting.

**Figure 2:**
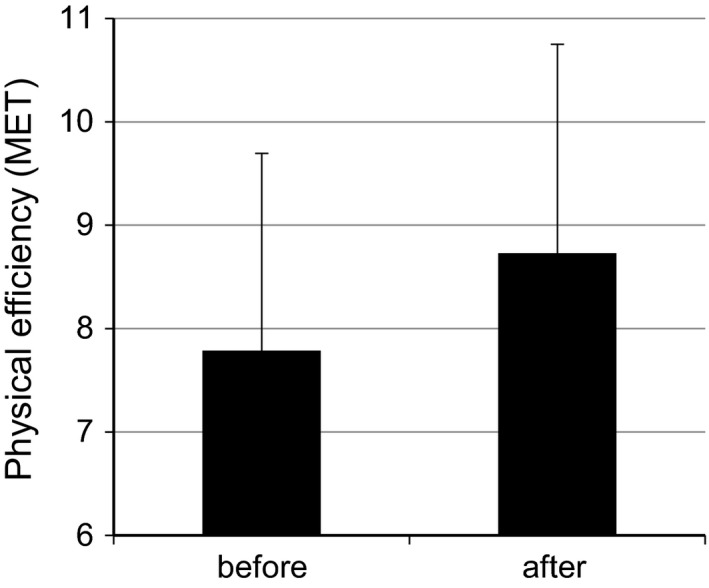
Physical efficiency of the patients in metabolic equivalents (before vs. after exercise therapy).

All 25 study participants finished the training program, and on average, 16.4 of 18 possible training units were performed. Table 1 shows a clearly diminished aerobic capacity of the inpatients with MDD compared with the control group in both the aerobic (VAT) and anaerobic (VO_2peak_, Power_max_) measures. During the initial constant load test (Table 2), all patients were in a lactate steady state, and the concentration of blood lactate was between 0.79 and 4.37 mmol/L. The subjective perceived exertion on the Borg scale was between 8 and 14. After the training program, a significant improvement in aerobic capacity was observed in the patient group for the incremental exercise (Fig. 1) and constant load tests (Table 2). Except for the VAT, none of the parameters of aerobic capacity were significantly different from those of the control group after the exercise therapy. The physical efficiency measured in METs improved significantly in the patient group (Fig. 2).

**Table 1:** Anthropometric data and results of the spiroergometry for patients (*n* = 25) and controls (*n* = 25)

Parameters	Patients		*P*	Cohen's *d*	Controls
	Before	After			
Age (Years)	45.4 ± 9.1				45.4 ± 10.6
Height (cm)	173 ± 10				174 ± 9
Weight (kg)	79.8 ± 21.9	78.9 ± 20.4	0.04	0.04	80.2 ± 16.0
BMI (kg/m^2^)	26.4 ± 5.3	26.2 ± 4.9	0.04	0.05	26.4 ± 3.9
RR_sys Rest_ (mmHg)	116 ± 14	117 ± 14	0.89	–	120 ± 15
RR_dia Rest_ (mmHg)	80 ± 10	79 ± 10	0.52	–	83 ± 8
HR_Rest_ (beats/min)	71 ± 13	71 ± 11	0.85	–	78 ± 14
Power_max_ (W)	161 ± 43	178 ± 44	0.00	0.38	195 ± 62^a^
Power_max_ (W/kg)	2.1 ± 0.5	2.3 ± 0.5	0.00	0.43	2.5 ± 0.8^a^
VO_2peak_ (mL/min)	2024 ± 582	2173 ± 565	0.02	0.26	2423 ± 690^a^
VO_2peak_ (mL/min/kg)	25.9 ± 6.5	28.1 ± 6.0	0.01	0.35	30.8 ± 8.8^a^
VAT (W)	62 ± 20	71 ± 22	0.00	0.42	91 ± 35^a^^,^ b
Borg	18.0 ± 0.9	17.7 ± 1.3	0.35	–	17.6 ± 1.4
HR_max_ (S/min)	164 ± 13	166 ± 14	0.27	–	169 ± 18
Lactate_max_ (mmol/L)	7.7 ± 1.5	8.1 ± 1.4	0.29	–	9.3 ± 3.2^a^
RR_sys max_ (mmHg)	181 ± 22	188 ± 25	0.08	–	192 ± 38
RR_dia max_ (mmHg)	91 ± 11	92 ± 9	0.82	–	93 ± 17
HR_Reserve_ (S/min)	93 ± 16	98 ± 16	0.14	–	83 ± 23^b^

BMI, Body Mass Index; HR, heart rate; RR, blood pressure; VO_2peak_, peak oxygen uptake; VAT, ventilatory anaerobic threshold.

a *P* < 0.05 Depression before versus Controls.

b *P* < 0.05 Depression after versus Controls.

**Table 2:** Mean values of the last 10 min of the training phase in the constant load test (with 80 ± 22 W) of the patients (*n* = 25)

Parameters	Patients		*P*	Cohen's *d*
	Before	After		
HR (beats/min)	124 ± 9	119 ± 10	0.04	0.53
RR_sys_ (mmHg)	142 ± 16	133 ± 13	<0.01	0.62
RR_dia_ (mmHg)	77 ± 5	74 ± 7	0.03	0.45
Lactate (mmol/L)	2.4 ± 0.9	1.9 ± 0.9	<0.01	0.62
Borg	12.0 ± 1.7	10.0 ± 2.2	<0.01	1.03

HR, heart rate; RR, blood pressure.

## Discussion

Hollenberg et al. (2003) found a reduced physical fitness and poorer results in a standardized treadmill test in women with depression who were above the age of 55 compared with a healthy control group, and this difference was independent of BMI. Boettger et al. (2009) reported a diminished physical fitness and delayed heart rate recovery as an indicator of a higher cardiovascular risk and autonomic dysfunction in 22 young patients (age 36.9 ± 13.1 years) with depression compared with a matched control group. Voderholzer et al. (2011) described an earlier and steeper lactate increase in patients with depression compared with healthy controls that was attributed to a worsened aerobic capacity. This was also apparent in our patient group because the VAT (which corresponds with the aerobic threshold, regardless of other measures) was reached earlier.

The results of our endurance test (Table 2) show a large range in the subjective perceived exertion, as well as in the objective load range, but without a significant correlation for both measures (*P* = 0.239). Nevertheless, all of the patients examined were in a lactate steady state, and this is an area that is suited for a long‐term training with patients. However, the load intensity that should be used in training therapy for patients with MDD remains controversial. With regard to the depressive symptomatology, Boettger et al. (2009) were able to demonstrate a correlation between the magnitude of the maximum lactate and an improvement in mood immediately after the patient reached physical exertion on a bicycle ergometer. This indicates a positive effect of highly intensive activity on depressive symptomatology.

Indeed, patients with depression often have problems integrating physical training into their everyday life and therefore need additional motivation (Voderholzer et al. 2011). There are considerations for beginning the training with intensities that are too high because of the reduced aerobic capacity, as this could lead to an excessive demand on the patients and preclude continuation in the training (Voderholzer et al. 2011). The training intensity we chose led to a significant improvement in aerobic and anaerobic capacity (in addition to a significant increase in the maximum load test, the values in the constant load test also were significantly improved) with satisfactory values on the Borg scale. Thus, this training program can be recommended for inpatients with MDD.

In a review, Warburton et al. (2006) described an increased mortality dependent on a reduced exercise capacity (measured in metabolic equivalent of task/MET), regardless of the basic illness, in which the group with an exercise capacity of >8 METs showed the lowest mortality. Our patient group improved from an intermediate risk (5–8 METs) to a low‐risk (>8 METs) group. Several studies have reported that physical training positively affects depressive symptomatology and quality of life in mild, moderate, and severe depression (Cooney et al. 2013; Silveira et al. 2013). Our data indicate that training included as a part of a multidisciplinary therapy can produce positive results in aerobic capacity during inpatient treatment.

However, a variety of factors, including reduced motivation, listlessness, decreased self‐esteem, and psychosomatic complaints, impede the initiation and continuation of exercise therapy. To ensure a permanent participation in training, additional motivation for behavior‐changing measures is necessary for patients with MDD (Prochaska and Velicer 1997; Miller and Rollnick 2002; Knapen et al. 2014). The program accomplished in our department considered important points, such as individual training adapted to the patient's objective physical capacity, and motivation with simultaneous supervision. The exercise therapy achieved a high acceptance rate, and over 90% of the possible training units were completed. The possibility of training in our department after dismissal from the inpatient treatment is available to the patients.

## Conclusion

The primary result of this study was that aerobic capacity in inpatients with MDD is improved by a structured supervised training program. After the intervention, parameters of aerobic capacity were comparable to healthy controls, except for VAT. Regarding the coincidence of depressive illnesses with cardiovascular and cardiometabolic disorders, a structured training program carried out during inpatient treatment is a meaningful addition to the standard therapy of depressed patients. Integration of physicians experienced in sports medicine, sports scientists, and physiotherapists is useful in the implementation of individually tailored training programs for patients with MDD.

### Limitation

The main limitation of the study was the small number of patients. Future studies should therefore take place on a multicentric basis and include larger numbers of patients in order to improve the general applicability of the results through use of a representative sample.

Because of the absence of a control group for the training intervention, training‐specific effects cannot be differentiated clearly from intervention‐bound effects.

## Conflict of Interest

None declared.
